# Cholangitis and Congestive Heart Failure Secondary to Biliary Hemorrhage in Hereditary Hemorrhagic Telangiectasia

**DOI:** 10.7759/cureus.75232

**Published:** 2024-12-06

**Authors:** Hiroki Yamamoto, Masamichi Kimura, Yumi Otoyama, Jun Imamura, Kiminori Kimura

**Affiliations:** 1 Hepatology, Tokyo Metropolitan Cancer and Infectious Diseases Center, Komagome Hospital, Tokyo, JPN

**Keywords:** abdominal pain, cholangitis, congestive heart failure, early detection, hepatic arteriovenous malformations, hereditary hemorrhagic telangiectasia

## Abstract

This case report discusses the case of a 74-year-old man who was diagnosed with hereditary hemorrhagic telangiectasia (HHT). The patient initially presented with right upper quadrant abdominal pain and was later diagnosed with cholangitis. Subsequently, heart failure was identified due to hepatic arteriovenous malformations. Although the exact duration of his disease remains unclear, the patient was initially asymptomatic and developed significant complications over time, possibly reflecting the progressive nature of vascular malformations associated with HHT. This case underscores the necessity of regular imaging follow-ups to assess the progression of vascular malformations in various organs, highlighting the clinical significance of the early detection and management of complications in HHT.

## Introduction

Hereditary hemorrhagic telangiectasia (HHT) is an autosomal dominant genetic disorder characterized by abnormal vascular formations, with an estimated prevalence of 1 in 5,000 to 8,000 individuals [1-3]. This disorder is commonly associated with mutations in the ENG and ACVR1/ALK1 genes, classified as HHT type 1 (HHT1) and HHT type 2 (HHT2), respectively [2]. Patients typically present with recurrent epistaxis, telangiectasias of the skin and mucous membranes, arteriovenous malformations (AVMs), and consequent iron deficiency anemia due to bleeding [3]. AVMs can occur in various organs, including the lungs, liver, and central nervous system, where pulmonary AVMs are particularly notorious for potentially causing life-threatening complications, such as brain abscesses and strokes, which are more commonly associated with CNS AVMs [3].

The liver is one of the organs most commonly affected by HHT and is notable for its complex vascular architecture [4-6]. Although the symptoms of hepatic vascular malformations (VMs) are often subtle, they can progressively worsen, leading to high-output cardiac failure (HOCF) and portal hypertension [7,8]. In addition, biliary diseases associated with HHT have been reported, including cholestasis and cholangitis, which are common complications. In addition, biliary diseases associated with HHT have been reported, including cholestasis and cholangitis, which are common complications [9-14].

Herein, we present and discuss the case of a patient who developed cholangitis and heart failure, both of which are complications of HHT. This case highlights the intricate interplay between multiple organ systems in patients with HHT and underscores the need for a multidisciplinary approach to its management and treatment.

## Case presentation

**Figure 1:**
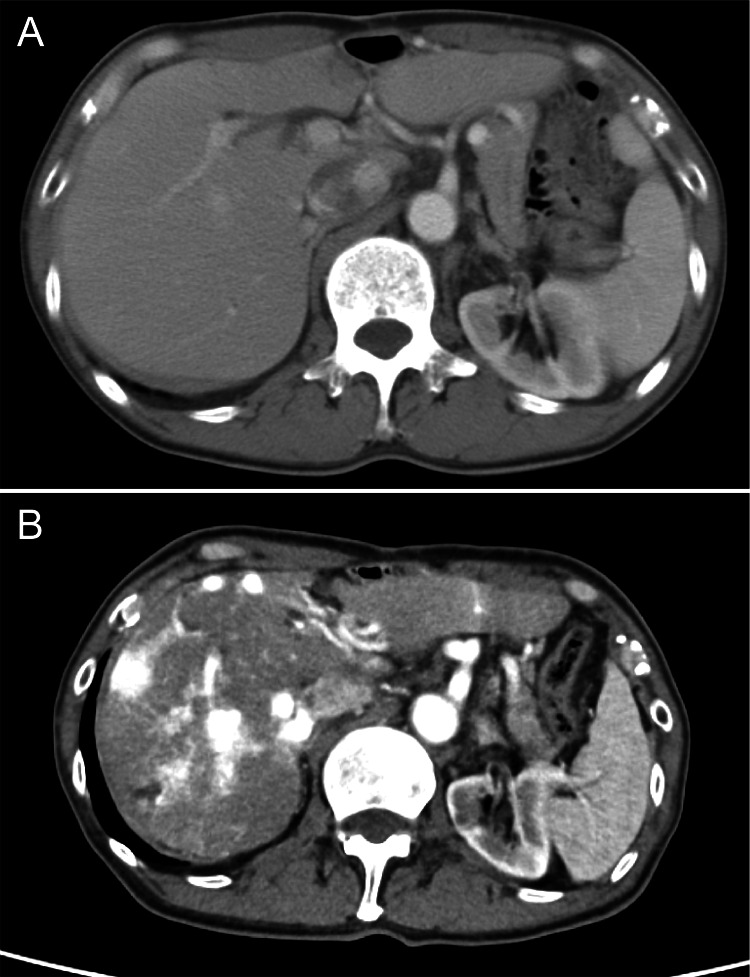
Contrast-enhanced computed tomography (CT) images of the abdomen (A) CT scan taken 20 years prior showing no abnormalities in the liver, highlighting a normal hepatic architecture without any notable lesions; (B) Recent CT scan demonstrating multiple arteriovenous malformations within the liver, illustrating the development of vascular abnormalities over time

A 74-year-old man with a history of esophageal cancer surgery in his 50s and no other significant medical history presented to a local physician with right-sided abdominal pain in June 2021. He was diagnosed with liver tumors in the right lobe following an abdominal ultrasound and contrast-enhanced computed tomography (CECT) scan. He was referred to our hospital for further examination. Twenty years before the consultation, he underwent surgery for esophageal cancer, and CT scans at that time revealed no hepatic lesions (Figure 1a). However, CECT performed during consultation revealed multiple AVMs in the liver, suggesting that the lesions had developed over time (Figure 1b). Physical examination revealed dilated vascular lesions on the dorsal of both feet and family history revealed that his brother had a cerebral hemorrhage and hepatic tumors.

Based on these findings, two of the Curaçao criteria were met, and the patient was diagnosed as having HHT. The Curaçao criteria for HHT diagnosis include spontaneous epistaxis; telangiectasias at specific sites (such as the lips, tongue, and fingers); visceral lesions (including pulmonary, hepatic, and cerebral AVMs); and a family history of HHT (Table 1).

**Table 1:** Diagnostic criteria for hereditary hemorrhagic telangiectasia (HHT)

Criteria	Definition
Clinical criteria	
Epistaxis	Spontaneous, recurrent nosebleeds
Telangiectases	Multiple at characteristic sites (lips, oral cavity, fingers, nose)
Visceral involvement	Pulmonary, liver, cerebral, spinal or GI vascular malformations
Family history	A first-degree relative with definite HHT
Diagnostic criteria	
Definite HHT	If 3 or 4 criteria are present
Probable HHT	If 2 criteria are present
HHT unlikely	If only 1 criterion is present

There was no history of epistaxis or brain or lung lesions. Moreover, genetic testing for ENG, ACVRL1, and SMAD4 mutations yielded negative results. The patient was followed up every three to six months after the initial presentation.

**Figure 2:**
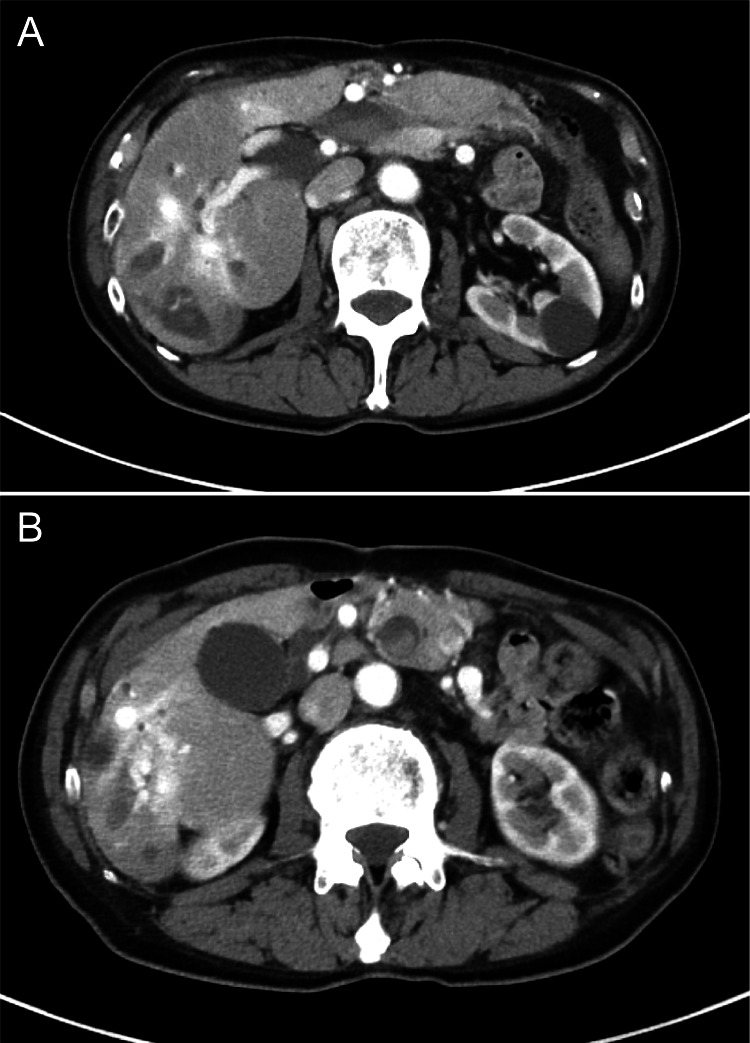
Contrast-enhanced computed tomography (CT) images showing hemobilia and hematoma (a) CT scan of the S6 segment of the liver, indicating suspected bleeding within the bile ducts, characterized by high-density areas suggesting active hemorrhage; (b) Detailed image of the common bile duct showing the presence of a hematoma visualized as an encapsulated high-density region within the ductal structure

**Figure 3:**
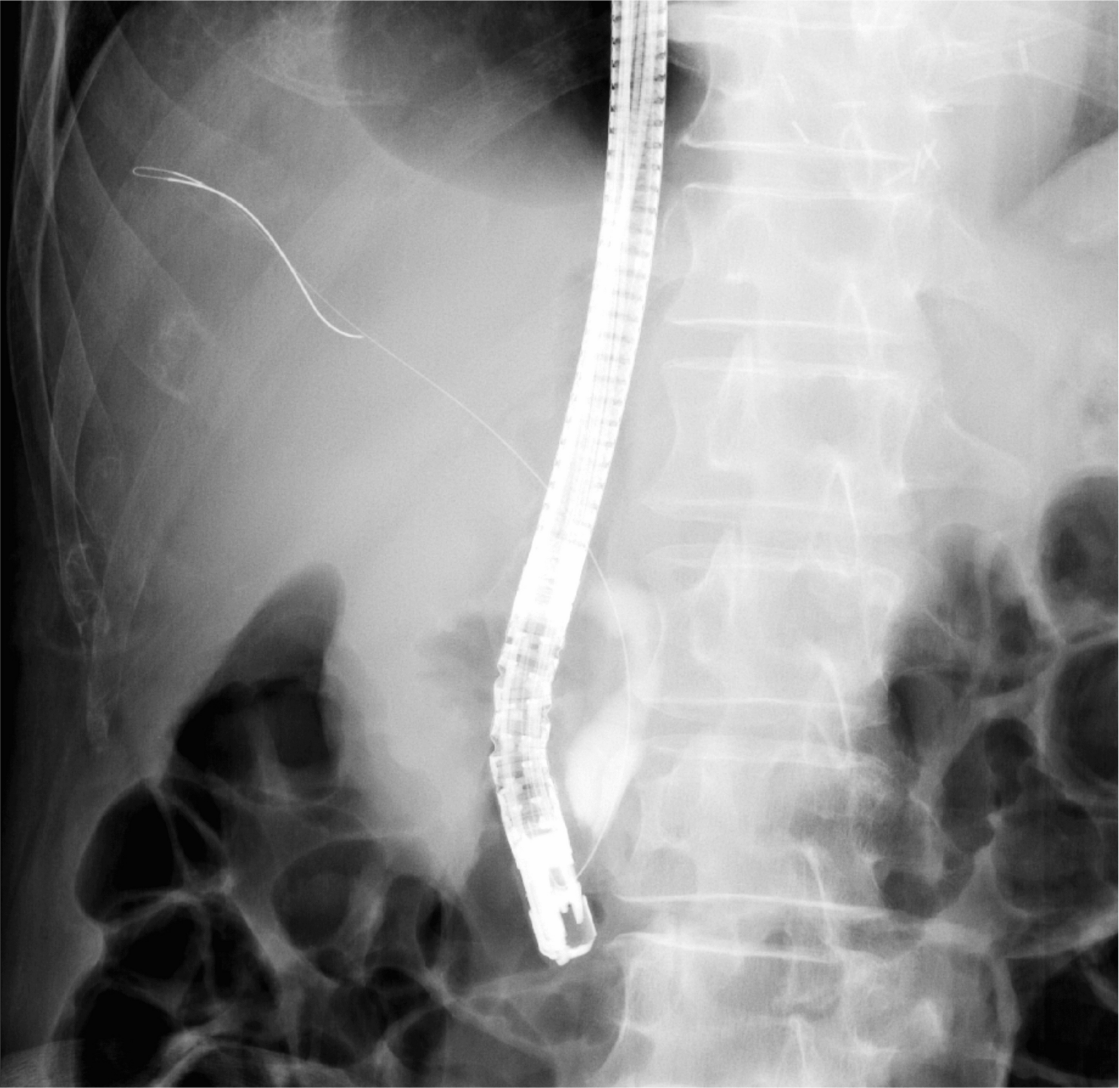
Endoscopic retrograde cholangiopancreatography image showing dilation of the common bile duct. This image captures the pronounced dilation of the common bile duct, indicative of obstructive processes, possibly due to intraductal pressure from an existing hematoma.

At the age of 76 years, in May 2023, the patient visited our hospital urgently because of sudden right upper quadrant abdominal pain. Upon admission, he had a fever of 39.9 °C, chills, and rigors. Blood tests indicated elevated inflammatory responses, mild anemia, and increased levels of hepatobiliary enzymes (Table 2). Abdominal contrast-enhanced CT revealed bleeding within the bile ducts in the S6 region of the liver and a hematoma in the common bile duct (Figures 2A, 2B). Endoscopic retrograde cholangiopancreatography revealed dilatation of the common bile duct (Figure 3). He was diagnosed with obstructive cholangitis due to an intrabiliary hematoma and was treated using antibiotics and biliary drainage. His symptoms improved after two weeks, and he was discharged.

**Table 2:** Results of the laboratory blood examination on admission WBC: white blood cell, RBC: red blood cell, Hb: hemoglobin, Plt: platelet, PT-INR: prothrombin time-international normalized ratio, BUN: blood urea nitrogen, Cre: creatinine, AST: aspartate aminotransferase, ALT: alanine aminotransferase, LDH: lactate dehydrogenase, ALP: alkaline phosphatase, γ-GTP: γ-glutamyl transferase, T-Bil: total bilirubin, D-Bil: direct bilirubin, CRP: C-reactive protein

Parameter	Value	Normal Range	Unit
WBC	11,500	3,300–8,600	/μL
RBC	340×10^4	435–555×10^4	/μL
Hb	10.6	13.7–16.8	g/dL
Plt	14.7×10^4	15.8–34.8×10^4	/μL
PT-INR	1.18	0.85–1.15	
Albumin	3.4	3.8–5.2	g/dL
BUN	17	8–20	mg/dL
Cre	0.89	0.65–1.07	mg/dL
AST	71	13–30	U/L
ALT	32	10–42	U/L
LDH	304	124–222	U/L
ALP	268	38–113	U/L
γ-GTP	238	13–64	U/L
T-Bil	1.5	0.4–1.5	mg/dL
D-Bil	0.7	0–0.4	mg/dL
Na	141	138–145	mmol/L
K	3.9	3.6–4.8	mmol/L
CL	106	101–108	mmol/L
CRP	6.70	0-0.14	mg/dL

**Figure 4:**
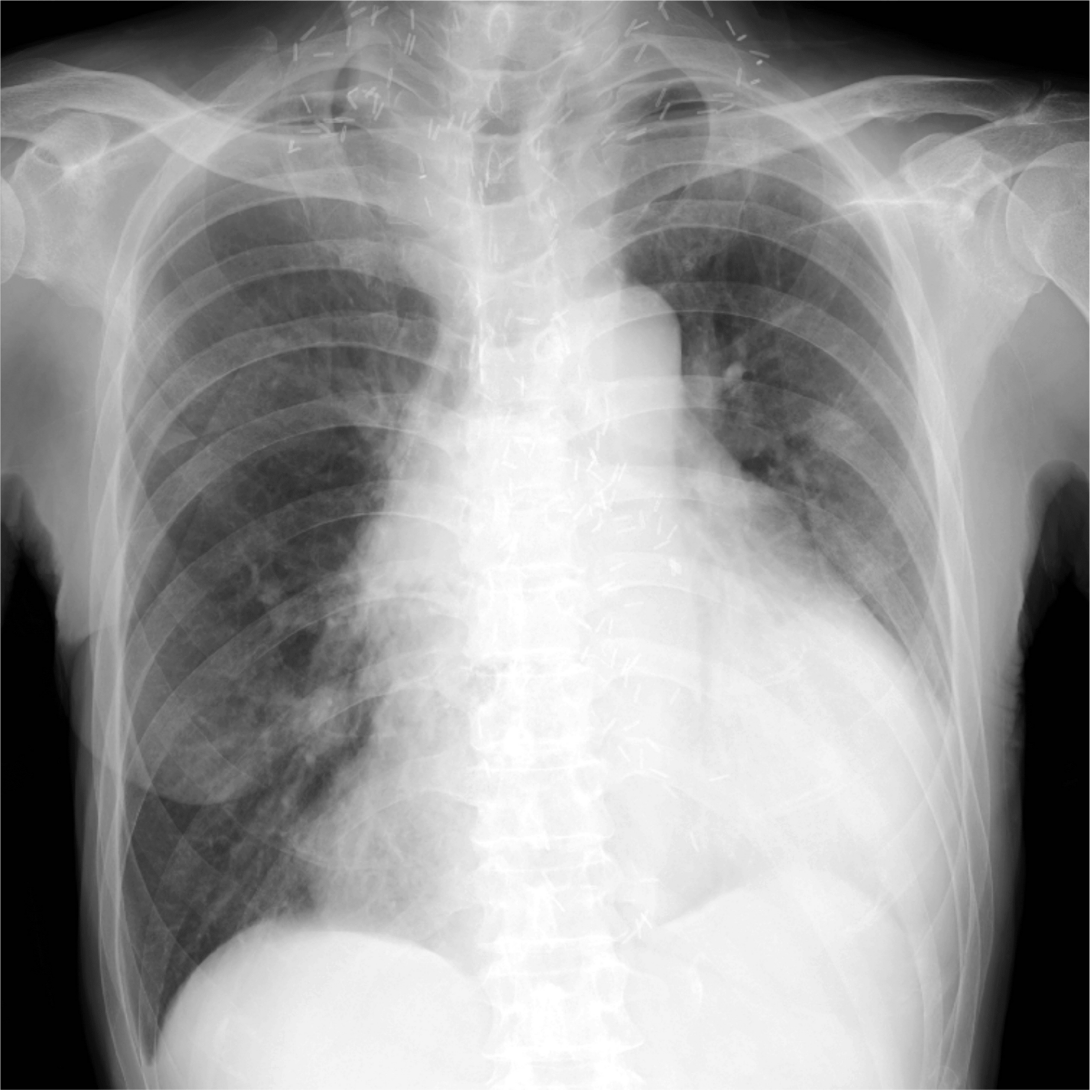
Chest radiography showing cardiac and pulmonary features. The chest radiograph highlights a cardiothoracic ratio of 69%, indicating cardiac enlargement. Both costophrenic angles appear dull, suggesting pleural effusion, consistent with the patient’s symptoms of congestive heart failure.

Approximately 1 month after discharge, the patient was readmitted owing to lower leg edema and shortness of breath. Chest radiography revealed bilateral pleural effusion and cardiac enlargement (Figure 4). Echocardiography revealed an ejection fraction of 48% with diffuse mild hypokinesis of the left ventricular wall, which was more consistent with heart failure due to hepatic arteriovenous malformations rather than HOCM. No structural abnormalities typical of HOCM, such as asymmetric septal hypertrophy, were observed. He was administered furosemide and spironolactone for congestive heart failure. During hospitalization, coronary angiography was performed to evaluate myocardial ischemia, which showed no significant stenosis of the coronary arteries. Based on these findings, it was speculated that the presence of large arteriovenous fistulas in the liver placed a significant burden on the heart, leading to heart failure. The patient had previously undergone surgery for esophageal cancer in his 40s but did not receive chemotherapy at the time. Therefore, the development of congestive heart failure (CHF) was most likely related to hepatic arteriovenous malformations, rather than chemotherapy-induced cardiotoxicity. The patient was discharged a week later as heart failure symptoms improved with the adjustment of diuretics and the introduction of bisoprolol.

Subsequent repeated episodes of acute cholangitis ultimately led to the placement of an endoscopic biliary drainage tube, after which the patient experienced no symptom recurrence. For heart failure, conservative therapy continued, with good control achieved by adjusting the doses of diuretics (furosemide and tolvaptan) and β-blockers (bisoprolol).

## Discussion

HHT is diagnosed using the Curaçao criteria, which assesses four key elements: spontaneous epistaxis, specific-site telangiectasia, visceral lesions, and a family history suggestive of HHT [15]. In this case, the patient met the “probable” criteria based on the presence of telangiectasia and liver AVMs. Despite suggestive familial indications, detailed information was unavailable, and no genetic mutations were identified. Genetic testing for the ENG, ACVRL1, and SMAD4 mutations is commonly performed in patients suspected of having HHT. While mutations in ENG and ACVRL1 are frequently identified in patients with HHT, negative results do not exclude the diagnosis, as a subset of patients with HHT do not carry detectable mutations [16]. This aligns with reports suggesting that 20% of clinically diagnosed HHT cases lack identifiable mutations, underlining the necessity for clinical vigilance even without genetic confirmation [16].

Although the prevalence of hepatic lesions in patients with HHT ranges from 41% to 78% [4-6], only approximately 8% of patients with radiologically apparent venous malformations (VMs) report their symptoms [4,5], with HOCF and portal hypertension being the most common. The dual blood supply of the liver permits three types of shunts: arteriovenous, arterioportal, and portovenous. Heart failure is believed to develop because of decreased effective hepatic perfusion and increased oxygen demand, coupled with reduced systemic vascular resistance, which stimulates the sympathetic nervous system and renin-angiotensin-aldosterone system, culminating in HOCF [7,8]. Concurrent increases in pulmonary blood flow and left ventricular failure can increase atrial pressure, leading to atrial dilation and potential atrial fibrillation [8]. Conservative management helped control heart failure in this case.

Regarding biliary effects, a few reports suggest that ischemia induced by hepatic artery shunts can cause cholangitis. Biliary tract inflammation due to intrabiliary hematomas is rare. Our review revealed only seven reported cases of HHT in patients with biliary bleeding. Among these, just two involved cholangitis secondary to biliary hematomas [9-14].

A notable aspect of our case was the remarkably short interval between the appearance of symptoms due to liver VMs and the onset of heart failure. While findings in patients with HHT typically show age-dependency [17], once lesions appear, rapid exacerbation of complications is possible, necessitating increased surveillance and timely diagnostics. Abdominal pain in patients with HHT should prompt the consideration of cholangitis due to biliary hematoma, a rare but potentially severe condition that leads to sepsis.

The patient has remained stable with symptomatic treatment; however, progression remains possible. For poorly controlled heart failure, interventions such as hepatic artery embolization or ligation are considered, whereas liver transplantation is recommended as a definitive treatment, although it may not be suitable because of the patient’s age [18].

## Conclusions

This case report highlights the complex interplay between hepatic vascular malformations and heart failure in a patient with HHT. The patient developed cholangitis and heart failure, both significant complications associated with HHT. The development of heart failure was linked to large arteriovenous fistulas in the liver, which placed a significant burden on the heart, leading to high-output cardiac failure. Despite the negative results from genetic testing, the diagnosis of HHT was supported by meeting the Curaçao criteria. Early intervention, including diuretic adjustment and the use of β-blockers, helped in managing heart failure while repeated cholangitis episodes necessitated the placement of an endoscopic biliary drainage tube for symptom resolution.

The case underscores the importance of early diagnosis and a multidisciplinary approach to managing the various systemic complications associated with HHT, particularly the progression of hepatic vascular malformations and their impact on multiple organ systems. Given the potential for rapid progression of complications, increased surveillance and timely diagnostics are crucial for improving patient outcomes. While liver transplantation remains a definitive treatment option for severe cases, careful consideration of patient factors, such as age, is essential for determining the most appropriate therapeutic approach.
